# What’s behind Margin Status in Oral Cancer?

**DOI:** 10.1007/s12070-024-04943-x

**Published:** 2024-07-30

**Authors:** Marco Piovesana, Francesca Boscolo Nata, Nicoletta Gardenal, Margherita Tofanelli, Paolo Boscolo-Rizzo, Rossana Bussani, Giancarlo Tirelli

**Affiliations:** 1 Department of Otorhinolaryngology Head & Neck Surgery, ULSS 4 Veneto Orientale, Via Piemonte 1, Portogruaro, VE 30026 Italy; 2 Head and Neck Department, ENT Clinic, University of Trieste, Strada di Fiume 447, Trieste, Italy; 3 Department of Medical, Surgical and Health Sciences, Section of Otolaryngology, University of Trieste, Piazzale Europa 1, Trieste, 34127 Italy; 4 UCO Pathological Anatomy and Histopathology Unit, Azienda Sanitaria Universitaria Integrata di Trieste, Strada di Fiume 447, Trieste, Italy

**Keywords:** Oral Squamous cell carcinoma, Surgical margins, Local recurrence, Adjuvant treatment, Shrinkage, Optical imaging

## Abstract

In the 2nd century AD, Galen argued that the failure to remove any single ‘root’ of a malignant tumor could result in a local relapse. After nearly 2 millennia, this problem appears to be even more challenging due to our increased understanding of the complexity of tumor formation and spread. Pathological analysis of tumor margins under a microscope remains the primary and only accepted method for confirming the complete tumor removal. However, this method is not an all-or-nothing test, and it can be compromised by various intrinsic and extrinsic limitations. Among the intrinsic limitations of pathological analysis we recall the pathologist handling, tissue shrinkage, the detection of minimal residual disease and the persistence of a precancerous field. Extrinsic limitations relate to surgical tools and their thermal damage, the different kinds of surgical resections and frozen sections collection. Surgeons, as well as oncologists and radiotherapists, should be well aware of and deeply understand these limitations to avoid misinterpretation of margin status, which can have serious consequences. Meanwhile, new technologies such as Narrow band imaging have shown promising results in assisting with the achievement of clear superficial resection margins. More recently, emerging techniques like Raman spectroscopy and near-infrared fluorescence have shown potential as real-time guides for surgical resection.

The aim of this narrative review is to provide valuable insights into the complex process of margin analysis and underscore the importance of interdisciplinary collaboration between pathologists, surgeons, oncologists, and radiotherapists to optimize patient outcomes in oral cancer surgery.

## Introduction

Oral squamous cell cancers (OSCC) account for approximately 2.1% of all new cancer diagnosis in developed countries [1]. The standard treatment includes surgical tumour resection, possibly with cervical node dissection, followed by adjuvant radiotherapy (RT) or radio chemotherapy (RTCT) if necessary [2, 3]. The decision regarding adjuvant treatments depends on staging and high risk pathological characteristics [2–4]. These indications are based on dated multicentric and prospective reports from radiotherapists and oncologists, which considered all head and neck (HN) sites together [2, 3], and lack surgical and pathological details [5] which can significantly impact margins classification and the need for adjuvant treatments [6].

Since the 2nd century AD with Galen, complete tumour removal has been the primary goal for oncologic surgeons. Pathological analysis of margins under the microscope, measuring the millimetres (mm) between the invasive tumour front and the scalpel cutting line, is still the only accepted method for verifying complete tumour removal [6]. However, this method is far from standardized and objective, as it can be influenced by various intrinsic and extrinsic limitations. Furthermore, there is a lack of an absolute and universally agreed-upon pathological definition of a free margin.

In this review, we analysed these “brain teasers” for HN surgeons, with a particular focus on the latest techniques that could help overcome these limitations. We selected relevant articles from the recent English scientific literature on PubMed.

## Practical Consequences of Margins Status

The National Comprehensive Cancer Network (NCCN) guidelines categorize margins as clear (≥ 5 mm), close (less than 2–5 mm, depending on the anatomical site involved), or involved (tumour at the inked margin), based on the distance in millimetres (mm) between the invasive tumour front and the cutting margin [7].

These strict rules have direct consequences for treatment protocols and significantly impact patients’ prognosis and quality of life [8].

Positive or close margins require re-resection if feasible. If revision surgery is not possible due to a reconstructive flap in place or the patient’s unwillingness, adjuvant radiotherapy is considered. This recommendation is primarily based on the results of two multicentric prospective trials published in 2004: the Radiation Therapy Oncology Group (RTOG) 9501 trial and the European Organization for Research and Treatment of Cancer (EORTC) 22931 trial [2, 3]. Surprisingly, these studies included mixed groups of tumours originating from the oral cavity, oropharynx, hypopharynx, and larynx. Additionally, adjuvant radiotherapy was indicated for positive margins in RTOG and for both close and positive margins in EORTC.

It is worth noting that these relatively dated studies were primarily conducted by radiotherapists and oncologists, and they did not provide details, even in a rough sense, regarding margin sampling, histopathological analysis, surgical approach, or the surgical tools used.

However, as mentioned earlier, the reliability of pathological analysis of surgical margins is affected by several factors, which, in our opinion, can be classified as intrinsic (handling of the samples by the pathologist, tissue shrinkage, and limitations in detecting small clusters of neoplastic cells), and extrinsic (surgical tools used by the surgeon with consequent thermal damage, surgical approach employed (en-bloc vs. piecemeal), and frozen sections sampling).

## Limitations of Pathological Analysis

### Intrinsic Limitations

**a) Pathologist handling**

After surgical removal, the specimen is fixed to preserve cell morphology and tissue architecture before being visually examined during the grossing step. The method of sectioning is debated in HN cancers, as there are no standardized procedures unlike other sites such as the colon or breast. Generally, specimens can be cut using the “radial or perpendicular” (right angle) or the “parallel or en-face” (shave margin) method. Only the first method allows for measuring the microscopic distance in millimetres between the inked margin and the tumour [9]. Despite the apparent linearity and simplicity of the grossing step procedures, it is important to highlight the potential for mistakes during the initial stages of sampling and the subjectivity that can exist among pathologists in the subsequent histologic interpretation [10].

Regardless of the method of grossing tumour used, each of the subsequent histological processing steps (dehydration, clearing and infiltration, microtomy cutting, haematoxylin and eosin staining, and placement on the microscope) theoretically has the potential to cause tissue alterations that can impact microscopic analysis [6].

Therefore, there is an urgent need for more concrete guidelines to better define the criteria for correct and standardized pathological margin assessment and reporting. Additionally, there is a general consensus on the specific need to establish a group of dedicated HN pathologists collaborating with surgeons from the time of surgical tumour removal through the initial stages of tissue processing. This collaboration could facilitate the development of specialized expertise and promote a closer working relationship between surgeons and pathologists to address the mutual challenges in interpreting these highly complex specimens.

**b) Tissue shrinkage**

The necessity to resect approximately 1–1.5 cm of normal tissue together with the tumour [7] is primarily due to the well-known phenomenon of shrinkage, which occurs immediately after resection and to a lesser extent after formalin fixation. It also appears to vary within different regions of a single specimen [6], less intra-tumoral and more pronounced in the marginal region: consequently, surgical margins tend to move closer to the tumour mass [11].

This raises the question: Is specimen shrinkage predictable? And how can we utilize this information in margin assessment? An interesting recent study by Burns et al. [12] found an average shrinkage of 26% (post-resection and post-processing) and suggested adjusting margin measurements accordingly. This proposal could potentially eliminate the need for larger resections in situations where they are unachievable due to tumours located in functionally or cosmetically important areas. Furthermore, it could help avoid the necessity for re-resection or adjuvant treatment in cases with close or positive margins. However, it should be noted that this remains a proposal at present.

**c) Minimal residual disease and precancerous field**

Even when surgical margins are histologically tumour-free, local recurrence may still occur in 10–30% of cases. These unexpected local relapses can be attributed to two different mechanisms: minimal residual disease (MRD) and the persistence of a precancerous field [13].

MRD refers to the persistence of small tumour cell clusters in the margins after surgery. These clusters may be too small to be identified through routine histopathology, making their detection in a single or a few pathological sections within a large tissue volume extremely challenging or even impossible. The presence of these unrecognized clusters along the cutting line can eventually lead to recurrence at the surgical site, even if the margin was initially classified as clear [13].

Immunohistochemistry (IHC) can aid in detecting residual isolated cells or small clusters, as used in sentinel node biopsies analysis [14].

Precancerous field, involves the persistence of genetically mutated cells that are phenotypically indistinguishable from normal cells. As a result, they are not visible to the naked eye during resection or histopathological margin analysis. Various techniques, including immunohistochemistry and the detection of copy number changes, have limited clinical use currently [13].

### Extrinsic Limitations

**a) Surgical tools and thermal damage of margins**

New surgical instruments using radiofrequency or ultrasound for cutting and haemostasis have emerged in the last decades, replacing cold blades, ligatures, or clips. However, all these instruments can potentially cause damage to nearby tissues due to the lateral spread of thermal energy. The extent of lateral thermal spread varies depending on the type of instrument, power setting, and application time [15].

Electrosurgical devices, such as the monopolar scalpel and bipolar forceps, are the most commonly used instruments in HN surgery. The monopolar scalpel remains the gold standard and is widely used worldwide, despite being shown to generate the highest temperatures and cause the greatest tissue damage, up to 1.5 cm [15]. On the other hand, newer generation instruments, such as the Electrothermal Bipolar Vessel Sealing System, Harmonic Scalpel, and CO2 Laser, produce minimal thermal damage (in the order of micrometres) [15, 16]. According to Mannelli et al. [17], thermal damage to specimens can lead to histopathological mistakes with potential therapeutic and prognostic implications. The possible consequences of thermal damage include: (i) false positives with indications for re-excision or adjuvant radiotherapy due to the loss of healthy tissue caused by tissue damage, resulting in a reduced readable distance between the margin and the tumour, or due to thermal changes that can mimic tumour characteristics, making it difficult to distinguish from cancer cells; (ii) false negatives with the absence of indications for re-excision or adjuvant radiotherapy, as thermal injury can partially destroy small cancerous or pre-cancerous cell clusters along the margin, falsely indicating complete resection. Thus, the extent of thermal injury caused by the instruments used for tumour resection is of utmost importance, as the pathologist cannot take into account the areas of epithelium affected by thermal injury [15–17].

It should be noted that new generation instruments were introduced in the early 2000s, while the trials that definitively established the need for adjuvant therapy in case of adverse features were based on patients surgically treated between 1994 and 2000 [2, 3]. It can be speculated that during those years, the most commonly used instrument (although not specifically mentioned in the methods section) was the monopolar scalpel, with the potential consequences mentioned above. One might wonder if the use of newer generation tools causing less thermal damage would have yielded the same results in those studies….

**b) Different kind of surgical resection and frozen section sampling**

Recent decades have seen a shift from the traditional “en-bloc” resection, which involves removing the tumour along with a margin of 1–2 cm of normal appearing tissue, to the so-called “piecemeal” resection [6, 18]. In classical en-bloc resection, margins are primarily assessed during the final pathological examination, and the pathologist may include the peripheral part of the tumour in the section to determine the microscopic distance in millimetres between the inked margin and the tumour front [9, 19]. On the other hand, in piecemeal resection, radicality is achieved step by step through the removal of additional tissue strips from the tumour bed until a tumour-free margin is obtained during frozen section (FS) analysis [6, 19]. In this type of surgery, it can be challenging for the pathologist to accurately assess the true distance between the tumour front and the inked margins, as the tumour is not always present in every tissue strip. Consequently, calculating the total length of the margins by adding the widths of multiple tissue strips seems to be impractical [5, 9].

Given these considerations, the strict metric assessment proposed by current guidelines appears difficult to apply to piecemeal resection, even though it is a well-established surgical technique.

FS offers real-time information on the completeness of tumour resection. Despite the widespread use of FS [20] some open problems still exist. First, point sample technique results in FS that may not be representative of the entire margins. On the contrary, if FS are collected as tissue strips for superficial margins and as a bowl for the deep ones, surgical margins can be examined in their entirety [21]. Moreover, it is not clearly defined which site is more correct to collect FS from, whether from the tumour bed or the surgical specimen. Some studies suggest that the specimen driven approach is more predictive for local control [22]; however, a recent survey by Bulbul et al. demonstrated an almost similar distribution between the two methods among the interviewed surgeons [20]. A survey by the American Head and Neck Society [23], although a bit dated, showed that 76% of the respondents collected FS from the surgical bed. The risk of this approach is the difficulty in relocating the site to enlarge after a positive FS. However, a precise surgical specimen orientation and continuous communication between the surgeon and pathologist could overcome this issue [21]. Finally, the prognostic impact of FS remains debated, with some studies showing worse outcomes for initially positive margins, regardless of whether re-resection guided by FS was performed [24], while others did not find such an association [25].

## Future Directions

As defined by Hinni et al. [19], surgical margin refers to any tissue plane where the surgeon’s knife meets the patient. This action primarily depends on the surgeon and represents a crucial step, as it has been proven that failure to achieve a clear surgical margin results in a higher risk of recurrence and decreased survival [25].

However, we are still far from defining a method that allows surgeons to precisely identify tumour margins intraoperatively. In the past fifteen years, optical imaging techniques have provided valuable tools in this regard. Among these techniques, narrow band imaging (NBI) has gained widespread use and has shown promising results in the literature. NBI utilizes light filters that allow the passage of only two specific wavelengths, highlighting the altered tissue vasculature seen in cancers. Initially used during follow-up [26], NBI has later been applied intraoperatively to identify cancerous and dysplastic areas in real time that can extend beyond the safety margins of 2 cm typically left by the surgeon, and are often not visible to the naked eye [18, 27]. NBI’s efficacy is supported by studies correlating NBI-guided margins with genetic analysis and reduced recurrence rates [28].

Additionally, other technologies like Raman spectroscopy and near-infrared fluorescence are being explored for margin assessment.

### Raman Spectroscopy

Raman spectroscopy (RS) appears to be one of the most promising techniques for detecting early cancer cells in tissues [29].

RS relies on the release of vibrational energy generated by the interaction between laser light and the atomic bonds of molecules. The amount of vibrational energy, analysed as a peak of wavelengths, characterizes a specific spectrum, which represents the biochemical fingerprint of a molecule [29].

Therefore, RS can identify biomolecular changes within cells as they transition from a healthy to a cancerous state. Tumours, including HN cancers, release metabolic products or biologically active molecules such as proteins, lipids, and nucleic acids into their environment (solid tissue, saliva, urine, or blood). This makes RS suitable for early cancer detection or analysis of tissue samples [30].

In the context of oral cavity carcinogenesis, Kumar et al. [31] demonstrated that healthy tissue and premalignant lesions exhibit increased levels of lipids compared to malignant specimens, where there is an increase in proteins and nucleic acids.

In terms of RS application in tissue sample analysis, Barroso et al. [32] found a high concordance between RS and haematoxylin eosin staining, indicating that RS is very promising for accurate diagnosis of cancerous tissue. However, its application in clinical practice has been limited by the long acquisition time (several hours for 1 mm^2^ of tissue) and the restricted field of view obtained with the intraoperative spectroscopic systems [33]. Recently, Horgan et al. [34] addressed these issues by presenting a new approach for acquiring spatial Raman spectroscopic diagnostic information using computer vision tracking of a handheld spectroscopic probe, where the data are overlaid onto the surgical field imaging to provide an augmented reality display.

### Fluorescence Guided Surgery

In the last decade, fluorescence-guided surgery (FGS) has become a booming research area, as it promises significant advancements in surgical oncology. It could enable a real-time guidance in delineating tumour boundaries during surgery using “simple” mobile cameras. Specifically, FGS utilizes fluorescent agents called “fluorophores,” which accumulate in tumoral tissue, causing it to fluoresce, while normal tissue does not [35]. The so called “tumour background ratio” (TBR) measures fluorescence accumulation in the tumour compared to the surrounding tissues. Tumour imaging visualization has been performed in an “open-field” setting, i.e., in the operating theatre before or during surgery, or in a “closed-field/back table” setting after surgery to analyse the margins of surgical specimens [36].

The most promising fluorescent agents are known as targeted near-infrared agents. These agents consist of fluorescent molecules attached to specific ligands such as peptides, antibodies, or small molecules like nanobodies. Targets that have been studied in HN squamous cell carcinoma include epithelial growth factor receptors (EGFR), vascular endothelial growth factor (VEGF), and metabolic tumour changes such as acidosis [37].

Among these targets, the use of EGFR has shown better results due to its high affinity with Cetuximab, which is a chimeric human-murine monoclonal antibody [38]. More recently, the safer and more specific human antibody Panitumumab [39] has also been used. Gao et al. [40] successfully demonstrated the value of fluorescence in guiding the collection of frozen sections, as they found that positive margins (i.e., <1 mm) exhibit more fluorescence than negative margins, both on the surface and in depth. The sensitivity and negative predictive value in relation to pathological findings were both 100%. However, FGS still faces challenges in open-field settings due to issues like ambient light interference and camera distance.

## Conclusions

This review aims to provocatively emphasize all the aspects that can affect margin analysis. In general, this intricate evaluation process is not well understood by clinicians and is often considered a simple piece of data.

By highlighting the numerous intrinsic and extrinsic factors that can influence margin analysis, we hope to increase awareness and promote enhanced collaboration between pathologists and surgeons. Surgeons should be mindful of the instruments used for resection to minimize thermal damage to the tissues. Furthermore, it is important for pathologists to have more defined guidelines for surgical specimens processing.

Moreover, our intentionally provocative comments are intended to stimulate reflection among radiotherapists and oncologists regarding the pre-treatment factors affecting adjuvant treatments choices. Although several technologies are theoretically applicable in this field, they are currently in the research phase. However, there is hope that in the near future, they can provide real assistance in the intraoperative determination of resection margins.
